# Long-term retinal cone rescue using a capsid mutant AAV8 vector in a mouse model of CNGA3-achromatopsia

**DOI:** 10.1371/journal.pone.0188032

**Published:** 2017-11-13

**Authors:** Xufeng Dai, Ying He, Hua Zhang, Yangyang Zhang, Yan Liu, Muran Wang, Hao Chen, Ji-jing Pang

**Affiliations:** School of Ophthalmology and Optometry, The Eye Hospital, Wenzhou Medical University, Wenzhou, Zhejiang, P.R. China; National Eye Institute, UNITED STATES

## Abstract

Adeno-associated virus (AAV) vectors are important gene delivery tools for the treatment of many recessively inherited retinal diseases. For example, a wild-type (WT) AAV5 vector can deliver a full-length *Cnga3* (cyclic nucleotide-gated channel alpha-3) cDNA to target cells of the cone photoreceptor function loss 5 (*cpfl5*) mouse, a spontaneous animal model of achromatopsia with a *Cnga3* mutation. Gene therapy restores cone-mediated function and blocks cone degeneration in the mice. However, since transgene expression delivered by an AAV vector shows relatively short-term effectiveness, this cannot be regarded as a very successful therapy. AAV2 and AAV8 vectors with capsid mutations have significantly enhanced transduction efficiency in retinas compared to WT AAV controls. In this study, AAV8 (Y447, 733F+T494V)-treated *cpfl5* retinas showed greater preservation of short-term cone electroretinogram (ERG) responses than AAV8 (Y447, 733F)- or AAV2 (Y272, 444, 500, 730F+T491V)-mediated treatments. To explore the long-term rescue effect, AAV8 (Y447, 733F+T494V)-treated *cpfl5* retinas were evaluated at 9 months following postnatal day 14 (P14) treatment. Rescued ERG responses in the cones of treated *cpfl5* eyes decreased with increasing age, but still maintained more than 60% of the WT mouse responses at the oldest time point examined. Expression of CNGA3 and M/S-opsins was maintained in cone outer segments of the treated *cpfl5* eyes and was equal to expression in age-matched WT retinas. Near-normal cone-mediated water maze behavior was observed in the treated *cpfl5* mice. As these are the longest follow-up data reported thus far, AAV8 with capsid Y-F and T-V mutations may be one of the most effective AAV vectors for long-term treatment in a naturally occurring mouse model of CNGA3 achromatopsia.

## Introduction

Achromatopsia, also known as rod monochromatism, is a relatively rare autosomal recessive retinal disorder characterized by cone photoreceptor dysfunction. Clinically, the disease is generally classified into complete (typical) and incomplete (atypical) forms [1]. Typical symptoms of complete achromatopsia are more severe than the incomplete form. They include seriously reduced visual acuity, nystagmus, photophobia, and color blindness [1]. With only rod-mediated vision, patients are extremely sensitive to light and have daylight blindness. To reduce photophobia, currently available medical care is to limit light exposure using dark glasses. With the development of adeno-associated virus (AAV) vectors as gene delivery tools for many recessively inherited retinal diseases, several promising gene therapy projects have been initiated [2–9]. Recent preclinical trials have made significant progress in providing effective treatment for achromatopsia. The first clinical trials of gene therapy are either underway or will be launched soon and they are expected to contribute important data on the safety and efficacy of these treatments [10].

Thus far, six genes have been implicated in achromatopsia-associated mutations [11–15]: cyclic nucleotide-gated channel alpha-3 (*Cnga3*) [16,17], cyclic nucleotide-gated channel beta-3 (*Cngb3*) [18,19], guanine nucleotide binding protein alpha transduction active peptide 2 (*Gnat2*) [1,20], phosphodiesterase 6C (*Pde6c*) [21,22], *Pde6h* [12,13], and cyclic AMP-dependent activating transcription factor-6 alpha (*Atf6*) [14,15]. The proteins encoded by these genes play vital roles in the phototransduction cascade of cone photoreceptors.

The *Cnga3* gene encodes a member of the cyclic nucleotide-gated ion channel protein family, which is critical for normal vision in cone photoreceptors [23]. As the first identified and second most common cause of achromatopsia, *Cnga3* mutations account for approximately 25% of all cases [24,25]. A cone photoreceptor function loss 5 (*cpfl5*) mouse strain, with a naturally occurring *Cnga3* mutation, was discovered at The Jackson Laboratory [11]. Due to a single nucleotide A to G transition at position 492 of exon 5, the deficient mice exhibit selective loss of cone-mediated electroretinogram (ERG) responses [11]. In addition, it has been shown that loss of CNGA3 results in impaired expression and trafficking of cone opsins [11,26].

New generations of viral vectors have made it possible to deliver functional genes to retinal cells [27]. Gene therapy, which can rescue visual function, has been used to treat achromatopsia in some animal models [28]. The most commonly used transgene vectors are those derived from AAVs. Early studies have shown short-term rescue [28,29]. In a later study using a wild-type (WT) AAV5 vector driven by the chicken beta actin (CBA) promoter, photopic ERG b-wave responses were maintained to an average of 80% of the WT mouse responses at 5 months following subretinal gene therapy [11]. However, a longer duration of rescue was not pursued in those studies. It cannot be considered a successful therapy if transgene expression delivered by an AAV vector shows only short-term effectiveness.

AAV vectors with different serotypes and capsid mutations have been developed. These include AAV2 and AAV8, with capsid surface-exposed tyrosine residues mutated to phenylalanine (Y-F) [11] and/or threonine mutated to valine (T-V) [30]. These mutations were shown to protect vector particles from proteasomal degradation [30–34]. Designing new vectors with these mutations may be an effective way to improve longevity of transgene expression.

In this study, three AAV vectors with different capsid mutations were compared for treatment of *cpfl5* mice. These vectors were AAV8 (Y447, 733F + T494V), AAV8 (Y447, 733F), and AAV2 (Y272, 444, 500, 730F + T491V). All vectors delivered the same *Cnga3* cDNA, but had different Y-F and/or T-V mutations on the capsid. We report the 9-month preservation of cone structure and function using the AAV8 (Y447, 733F + T494V) vector to deliver treatment in a mouse model of achromatopsia.

## Materials and methods

### Animals

The congenic inbred strain of the *cpfl5* mice and the isogenic WT C57BL/6J mice were acquired from The Jackson Laboratory (Bar Harbor, ME, USA). Mice were bred and maintained in the Animal Facilities of Wenzhou Medical University (Wenzhou, China). All animals were maintained on a cycle of 12 h of light and 12 h of dark, with free access to water and food. Animal experiments were approved by Wenzhou Medical University’s Institutional Animal Care and Use Committee (Permit Number: wydw2014-0072), and conducted according to the ARVO Statement for the Use of Animals in Ophthalmic and Vision Research.

### Construction of AAV vectors

The AAV8 (Y447, 733F + T494V) capsid is an AAV serotype 8 capsid with a double Y-to-F mutation at residue 447 and 733, accompanied by a single T-to-V mutation at residue 494. AAV8 (Y447, 733F + T494V) was used to package the vector DNA. For comparison, the vector DNA was also packaged in AAV8 (Y447, 733F) and AAV2 (Y272, 444, 500, 730F + T491V). IRBP/GNAT2 is a hybrid promoter consisting of a 277-bp GNAT2 promoter and a 214-bp interphotoreceptor retinoid binding protein (IRBP) enhancer. AAV vectors containing the IRBP/GNAT2 promoter exhibit cone photoreceptor-specific transgene expression [35]. Mouse *Cnga3* cDNA was cloned under the IRBP/GNAT2 promoter to make an AAV-IRBP/GNAT2-*Cnga3* construct [35]. All AAV vectors were constructed and purified at the University of Florida (Gainesville, FL, USA).

### Vector delivery via subretinal injection

Subretinal injection was performed at postnatal day 14 (P14) [36] to achieve maximum rescue of cone photoreceptors. *Cpfl5* mice were treated with AAV8 (Y447, 733F + T494V)-IRBP/GNAT2-*Cnga3* (Group 1), AAV2 (Y272, 444, 500, 730F + T491V)-IRBP/GNAT2-*Cnga3* (Group 2), or AAV8 (Y447, 733F)-IRBP/GNAT2-*Cnga3* (Group 3). One microliter of each vector solution (10^13^ vector genomes per mL) was injected subretinally into one eye of each *cpfl5* mouse. The other eye remained uninjected as a control. Subretinal injection was performed as described previously [36]. Each original AAV-vector solution (1E13 vg/ml) was diluted into 2E12 and 1E11 vg/ml, and the dilutions were also administered unilaterally by subretinal injection (1μl) on P14. A small amount of fluorescein (0.1 mg/mL final concentration) was routinely added to allow visualization of the AAV vector solution [36]. An injection was considered successful if blood vessels in the detached retina could be clearly seen with green dye underneath, suggesting that the AAV vector solution was in the subretinal space [36]. Mice were selected for further evaluation if they had minimal surgical complications and their initial detached retinal blebs (the area with vector solution underneath) covered more than 80% of the whole retina. We included at least six mice per group for statistical analysis.

### ERG recordings

After overnight dark adaptation, mice were anesthetized with a solution of ketamine (70 mg/kg) and xylazine (5 mg/kg) under dim red light. Full-field ERGs were recorded under a standard Ganzfeld dome, which is controlled by a computer-based system (Roland Consult, Wiesbaden, Germany). White light-emitting diodes (LEDs, 450–780 nm) were used as stimulation and background light sources. Scotopic ERGs were recorded at 0 log cd-s/m^2^ stimulus intensity [37]. With an interstimulus interval of 30 seconds, five responses were recorded and averaged. After adapting to a steady background illumination (30 cd/m^2^) for 10 min, photopic ERGs were recorded with a white-light stimulus intensity of +1.0 log cd-s/m^2^ [38]. To increase the signal:noise ratio, 50 individual ERG responses were averaged to produce the final waveform. The flash duration was set at 2 ms and the band pass of the amplifiers at 1–500 Hz. Amplitudes and peak times of ERG responses were saved for further evaluation.

### Immunohistochemistry

Mice were sacrificed by CO_2_ inhalation. Retinal sections were prepared as described in detail previously [11]. Briefly, eyes were enucleated and fixed immediately in 4% paraformaldehyde in 0.1 M phosphate buffer (pH = 7.4, 4°C) overnight. The cornea, lens, and vitreous were removed and the remaining eyecup was dehydrated in 30% sucrose for 4 h. After embedding in optimal cutting temperature medium (OCT; Sakura Finetek USA Inc., Torrance, CA, USA), samples were frozen in liquid nitrogen and cut on a cryostats. Cryosections (12-μm thick) were incubated overnight at 4°C with rabbit anti-mouse polyclonal CNGA3 primary antibody (1:200, bs-10772R; Bioss, Beijing, China). After three rinses with 0.1 M PBS, sections were incubated with goat anti-rabbit IgG conjugated to a Cy3 fluorochrome (1:400, AP187C; Merck Millipore, Darmstadt, Germany) for 2 h, followed by three rinses with 0.1 M PBS. Additionally, FITC-conjugated peanut agglutinin (PNA, 1:400; Vector Laboratories, Burlingame, CA, USA) was used to detect the interphotoreceptor matrix sheath, which surrounds the cone outer segments. Similarly, frozen sections were stained for M- or S-cone opsins [39]. Nuclei were stained with 4',6-diamidino-2- phenylindole (DAPI). Retinal whole-mounts were prepared and stained for M- or S-cone opsins as described previously [39]. Retinal cryosections and whole-mounts were mounted with coverslips and imaged by fluorescence microscopy.

### Visually guided water maze behavioral test

The visually guided water maze behavioral test was performed as described previously [40] with only minor modifications. Briefly, 9 months after injection, the AAV8 (Y447, 733F + T494V)-IRBP/GNAT2-*Cnga3*-treated *cpfl5* mice, together with age-matched untreated *cpfl5* and WT mice, were initially trained to escape to a small platform positioned randomly in a water tank. The water tank had a diameter of 1.2 meters. Before formal tests, mouse pupils were dilated (1% atropine) and retinas were fully light-adapted (100 cd/m^2^ for 10 min) to prevent rod intrusion. During each test, a mouse was initially placed in the water tank from one of four equally spaced starting locations. The time taken to escape to the randomly positioned platform was recorded as the visually guided behavioral data. If a mouse could not escape to the platform within 60 seconds, it was guided to the platform and its escape time was recorded as 60 seconds. The water maze tests were performed in well-lit (18 lux) environments.

### Statistical analysis

ERG data were presented as mean ± standard deviation (SD). SPSS 18.0 (IBM Corporation, Armonk, NY, USA) was used for statistical analysis. The data were checked by Shapiro-Wilk test for *normality before* applying any parametric test. Paired sample t-test or one-way ANOVA with least significant difference (LSD) post hoc test was used for data comparison between or among groups. A *P*-value of less than 0.05 was considered statistically significant.

## Results

### Rescue efficacy of AAV vectors with capsid Y-F and/or T-V mutations in *cpfl5* ERGs

**Fig 1:**
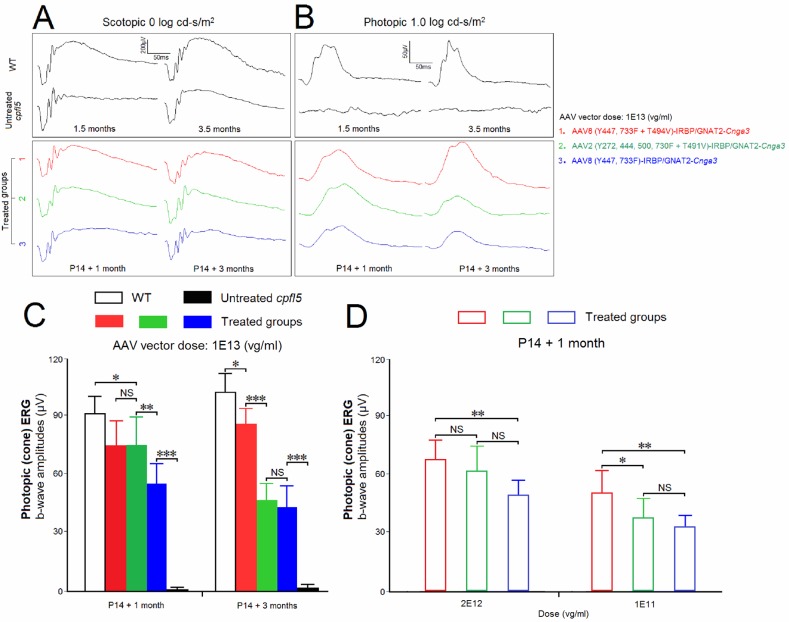
Comparisons of ERGs at two time-points following AAV vector treatment. As a mouse model of achromatopsia, *cpfl5* mice show selective loss of cone ERG responses. Scotopic (A) and photopic ERGs (B) were recorded at 1 and 3 months following treatment with AAV8 (Y447, 733F + T494V) (red), AAV2 (Y272, 444, 500, 730F + T491V) (green), or AAV8 (Y447, 733F) (blue). Recovered photopic ERG (cone-mediated) amplitudes were compared among groups (C). (D) Photopic-ERG dose response of the AAV-vector dilutions. The *cpfl5* mice were treated in one eye at P14 and evaluated at 1 month after subretinal injection. Age-matched WT and untreated *cpfl5* eyes were used as controls (n = 6 mice). P, postnatal day. *indicates *P* < 0.05, **indicates *P* < 0.01, ***indicates *P* < 0.001, NS = no statistical difference.

*Cpfl5* mice exhibit selective loss of cone ERG responses, similar to the electroretinographic phenotype of complete achromatopsia patients with *Cnga3* mutations. After treatment with one of the three AAV vectors at P14, eyes of *cpfl5* mice were tested by scotopic and photopic ERGs (Fig 1A and 1B). ERG data in each group were normally distributed (Shapiro-Wilk test, n = 6, *P* > 0.05). The photopic b-wave amplitudes were significantly improved in the three treated *cpfl5* groups (versus the untreated *cpfl5* eyes, *P* < 0.001), to 42%∼84% of amplitudes in age-matched WT mice (*P* < 0.05). Among the three treated groups, *Cnga3* was delivered by AAV8 (Y447, 733F + T494V), AAV2 (Y272, 444, 500, 730F + T491V), or AAV8 (Y447, 733F) vectors. At 1 month after treatment, early preservation of photopic ERG b-waves was statistically higher in AAV8 (Y447, 733F + T494V) (75 ± 13 μV) and AAV2 (Y272, 444, 500, 730F + T491V) (76 ± 14 μV) groups, compared with the AAV8 (Y447, 733F) group (56 ± 11 μV, *P* < 0.01; Fig 1C). Here, vectors with both Y-F and T-V mutations led to better ERG rescue than vectors with only a Y-F mutation. At 3 months after treatment, photopic amplitudes of AAV2 (Y272, 444, 500, 730F + T491V)- and AAV8 (Y447, 733F)-treated retinas decreased to 47 ± 9 μV and 44 ± 11 μV, respectively, while the average amplitude of the AAV8 (Y447, 733F + T494V) group continued to rise slightly to 86 ± 8 μV. According to these short-term efficacy data, the AAV8 vector with both Y-F and T-V mutations rescued the best cone ERG responses (Fig 1C). In addition, a dose escalation study for each vector had been performed. *Cpfl5* retinas were analyzed by photopic ERGs at 1 month post-injection (Fig 1D). Among the three AAV mutants, a dose at 1E13 vg/ml produced the best improvement for b-wave amplitudes.

### Long-term (9 months) ERG rescue of AAV8 (Y447, 733F + T494V)-treated *cpfl5* eyes

**Fig 2:**
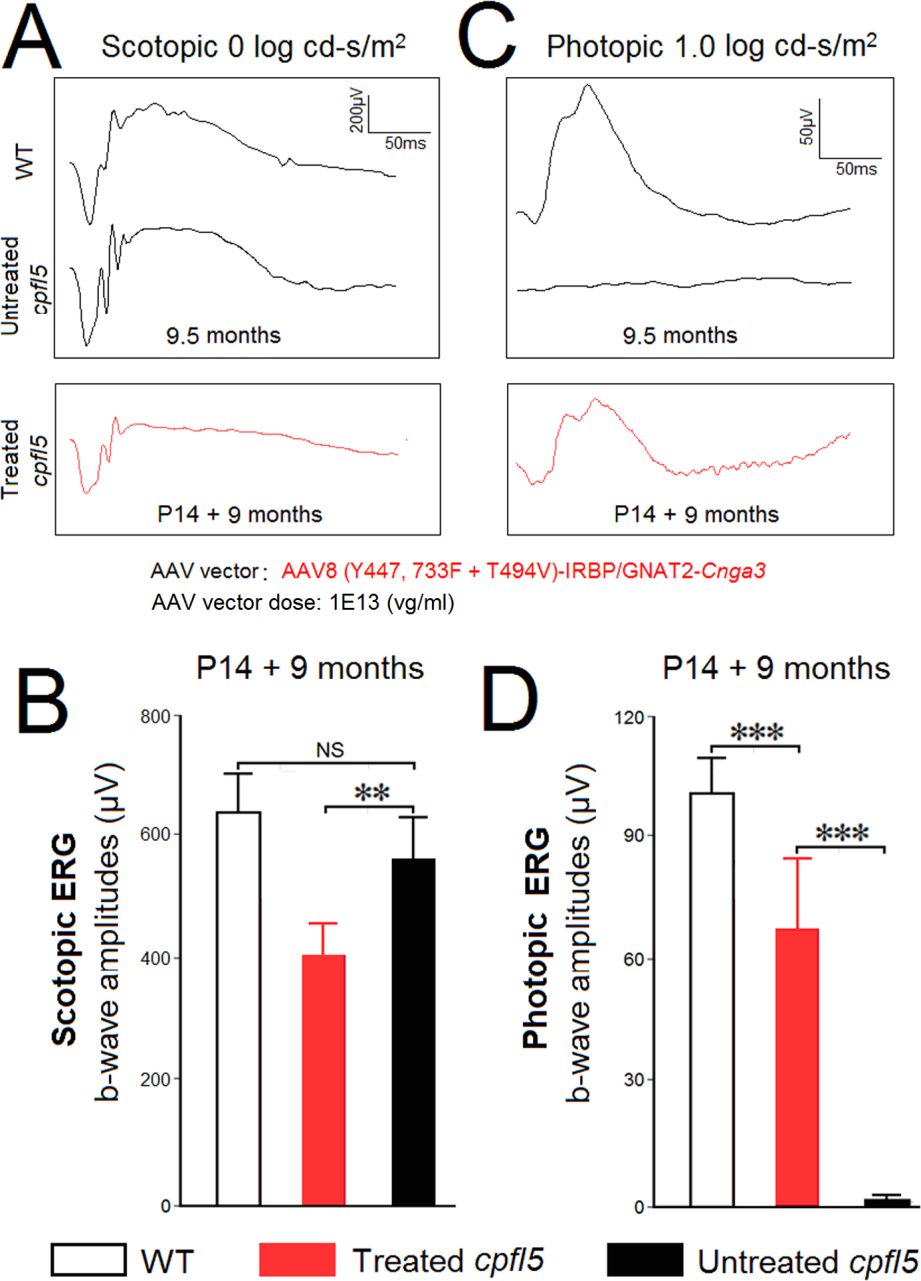
Long-term (9 months) electroretinographic assessment of treated *cpfl5* eyes. *Cpfl5* eyes were treated with AAV8 (Y447, 733F + T494V)-IRBP/GNAT2-*Cnga3* at P14. An ERG recording was repeated at 9 months following treatment. (A) Scotopic ERG elicited in treated *cpfl5* eyes (red), compared to WT and untreated *cpfl5* eyes (black). (B) Scotopic b-wave amplitudes elicited at 0 log cd-s/m^2^ intensity in the age-matched WT, treated, and untreated *cpfl5* eyes (n = 6). (C) Photopic ERG elicited in treated *cpfl5* eyes (red) compared to WT and untreated *cpfl5* eyes (black). (D) Photopic b-wave amplitudes elicited at 1.0 log cd-s/m^2^ intensity in age-matched WT, treated, and untreated *cpfl5* eyes (n = 6). P, postnatal day. **indicates *P* < 0.01, ***indicates *P* < 0.001, NS = no statistical difference.

Among the three AAV vectors, AAV8 (Y447, 733F + T494V)-IRBP/GNAT2-*Cnga3* was selected for long-term evaluation. Thus, an ERG recording was repeated in the 9.5-month-old *cpfl5* eyes. ERG data in each group were normally distributed (Shapiro-Wilk test, n = 6, *P* > 0.05). Untreated *cpfl5* (566 ± 70 μV) and WT eyes (643 ± 64 μV) had similar scotopic b-wave amplitudes (n = 6, *P* > 0.05; Fig 2A and 2B). However, there was a decrease in scotopic responses in treated eyes (410 ± 54 μV) compared with untreated eyes (*P* < 0.01; Fig 2A and 2B). Rescue of photopic ERG responses was detected in the treated *cpfl5* eyes, whereas ERG responses were nearly extinguished in the untreated *cpfl5* eyes (Fig 2C). At 9 months post injection, photopic ERG b-waves in the treated *cpfl5* maintained an average size of 68 ± 17 μV, about 65% of the WT level (101 ± 9 μV, n = 6, *P* < 0.001; Fig 2D). Furthermore, we analyzed implicit time of the rescued photopic b-wave. No significant difference was found between the treated *cpfl5* (60 ± 5 ms) and WT eyes (56 ± 3 ms, n = 6, *P* > 0.05).

### AAV-mediated CNGA3 expression in *cpfl5* retinas

**Fig 3:**
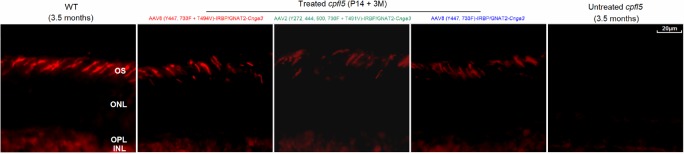
Immuno-staining of retinal CNGA3 at 3 months post-injection. CNGA3 expression (red) was detected primarily in the photoreceptor OS following treatment with AAV8 (Y447, 733F + T494V), AAV2 (Y272, 444, 500, 730F + T491V), or AAV8 (Y447, 733F). Age-matched WT and untreated *cpfl5* retinas were used as controls. OS, outer segment; ONL, outer nuclear layer; OPL, outer plexiform layer; INL, inner nuclear layer.

**Fig 4:**
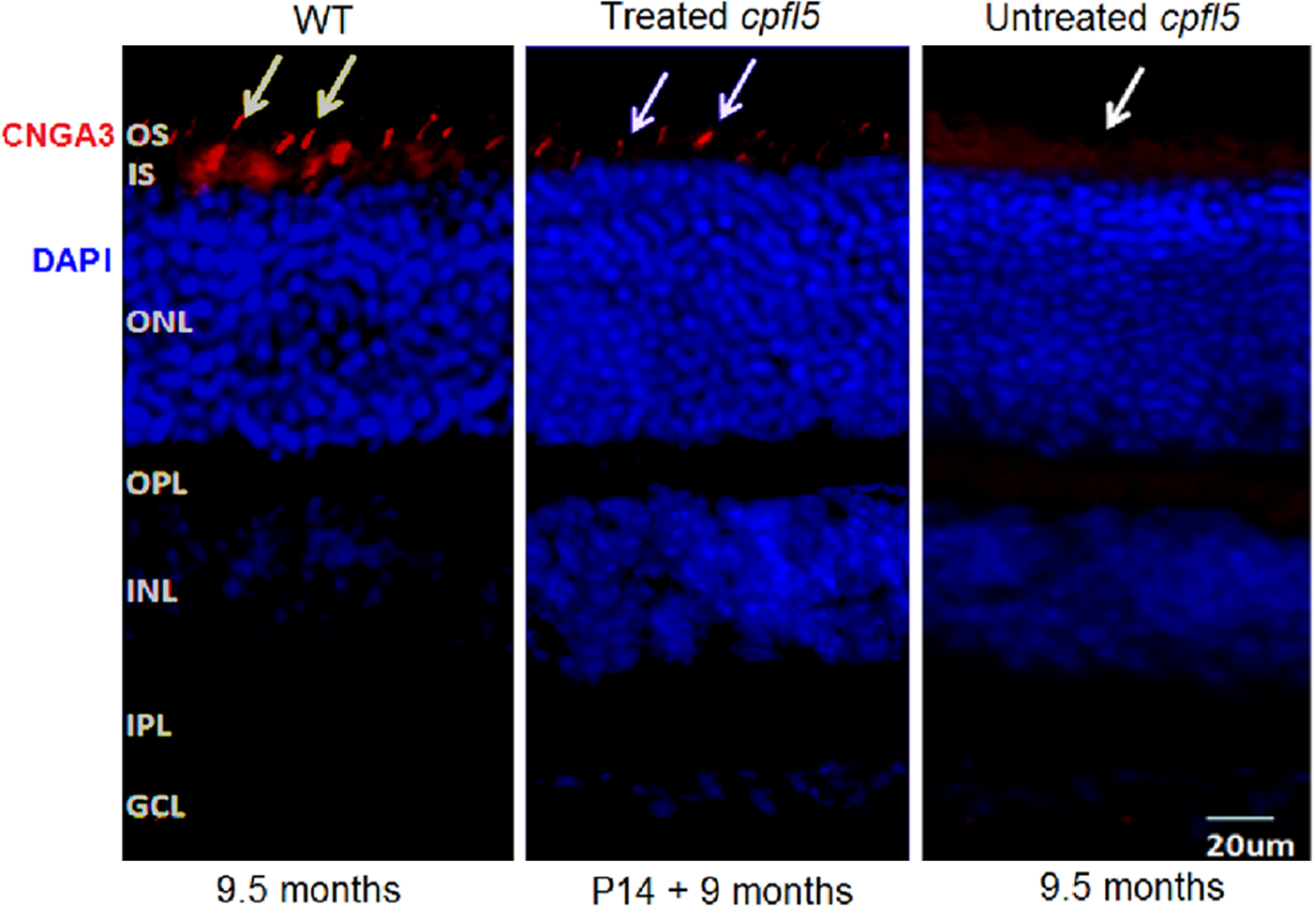
Long-term retinal CNGA3 expression mediated by AAV8 (Y447, 733F + T494V)-IRBP/GNAT2-*Cnga3* treatment. At 9 months following subretinal injection, retinal cryosections were immunostained with anti-CNGA3 antibody. CNGA3 expression (red) was detected primarily in the photoreceptor OS (arrows) in the AAV8 (Y447, 733F + T494V)-treated *cpfl5* retinas and age-matched WT controls, but not in the untreated *cpfl5* retinas. OS, outer segment; IS, inner segment; ONL, outer nuclear layer; OPL, outer plexiform layer; INL, inner nuclear layer; IPL, inner plexiform layer; GCL, ganglion cell layer. Nuclei were stained with DAPI (4′,6-diamidino-2-phenylindole) (blue).

Mediated by the Y-F and/or T-V mutant AAV vectors, cone-specific transgene expression was driven by the IRBP/GNAT2 promoter [35]. For maximum rescue of cone photoreceptors, subretinal injections were performed at P14. At 3 and 9 months after treatment, CNGA3 expression was assayed by immunohistochemistry. At 3 months following subretinal injection, CNGA3 staining was detected primarily in the photoreceptor outer segment (OS) layer of *cpfl5* retinas (Fig 3). Strong CNGA3 immunostaining was detected in the AAV8 (Y447, 733F + T494V)-treated group to a similar extent as in the age-matched WT retina. However, the immunostaining was relatively weaker in the AAV2 (Y272, 444, 500, 730F + T491V)- and AAV8 (Y447, 733F)-mediated groups. No CNGA3 expression was detected in the partner untreated retina from the same *cpfl5* mouse (Fig 3). In the AAV8 (Y447, 733F + T494V)-mediated group, transgene expression was also detected at 9 months after treatment (Fig 4). These are the longest follow-up data for CNGA3 expression thus far.

### Long-term rescue of cone opsins following AAV8 (Y447, 733F + T494V) treatment

**Fig 5:**
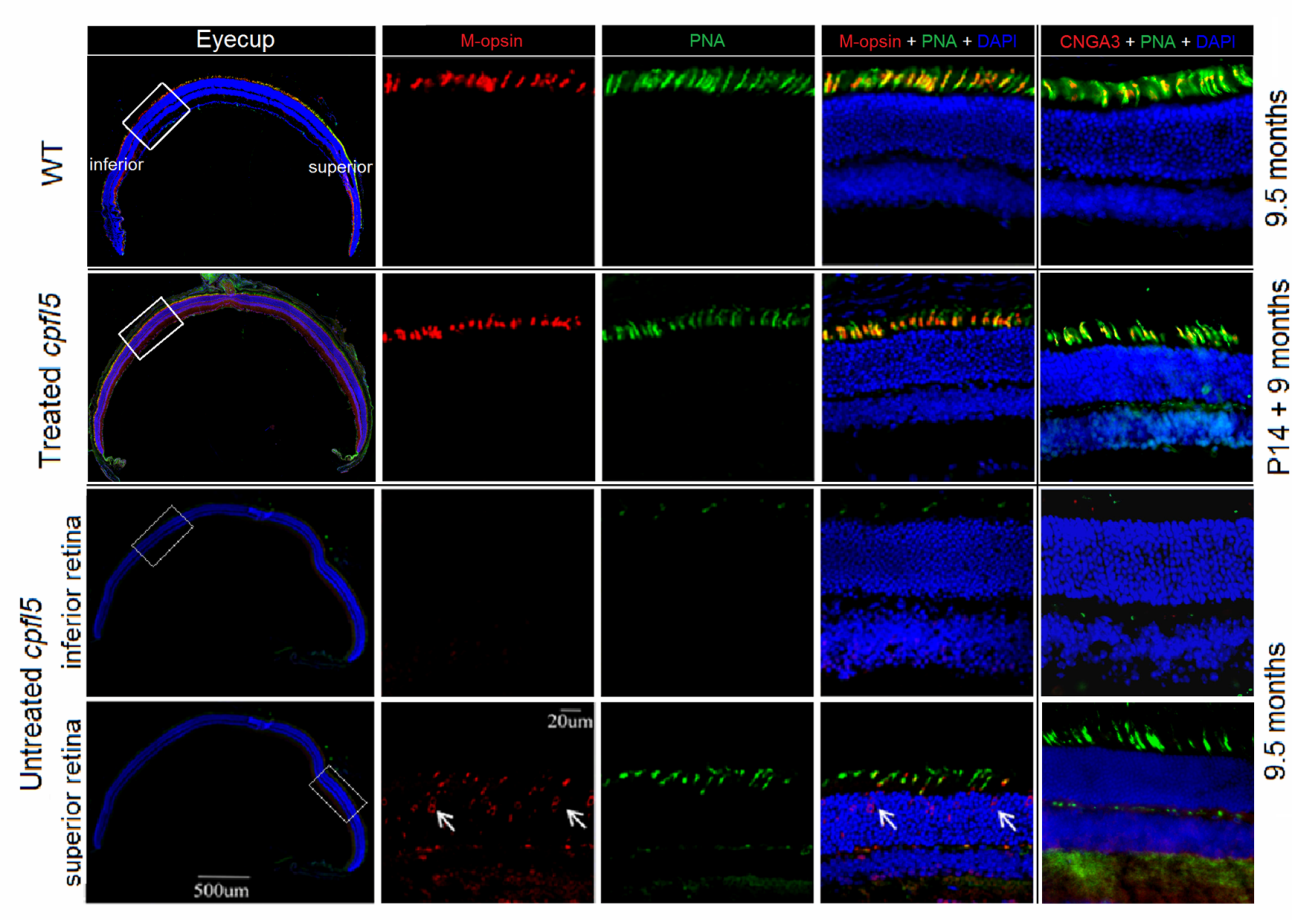
Long-term preservation of retinal M-opsin and CNGA3 after treatment with AAV8 (Y447, 733F + T494V)-IRBP/GNAT2-*Cnga3*. At 9 months after P14 treatment, *cpfl5* retinal immunostaining revealed normal expression and the cone OS distribution (merge) of M-opsin, compared with WT controls. In untreated *cpfl5* eyes, the inferior retinas had little M-opsin and cone specific PNA staining, whereas the superior retinas showed mislocalization of M-opsin within residue cones (arrows). In WT and treated *cpfl5* retinas, M-opsin was located in the cone OS. Red: M-opsin or CNGA3 staining; Green: cone-specific PNA staining; Blue: nuclei staining with DAPI (4′,6-diamidino-2-phenylindole). P, postnatal day.

**Fig 6:**
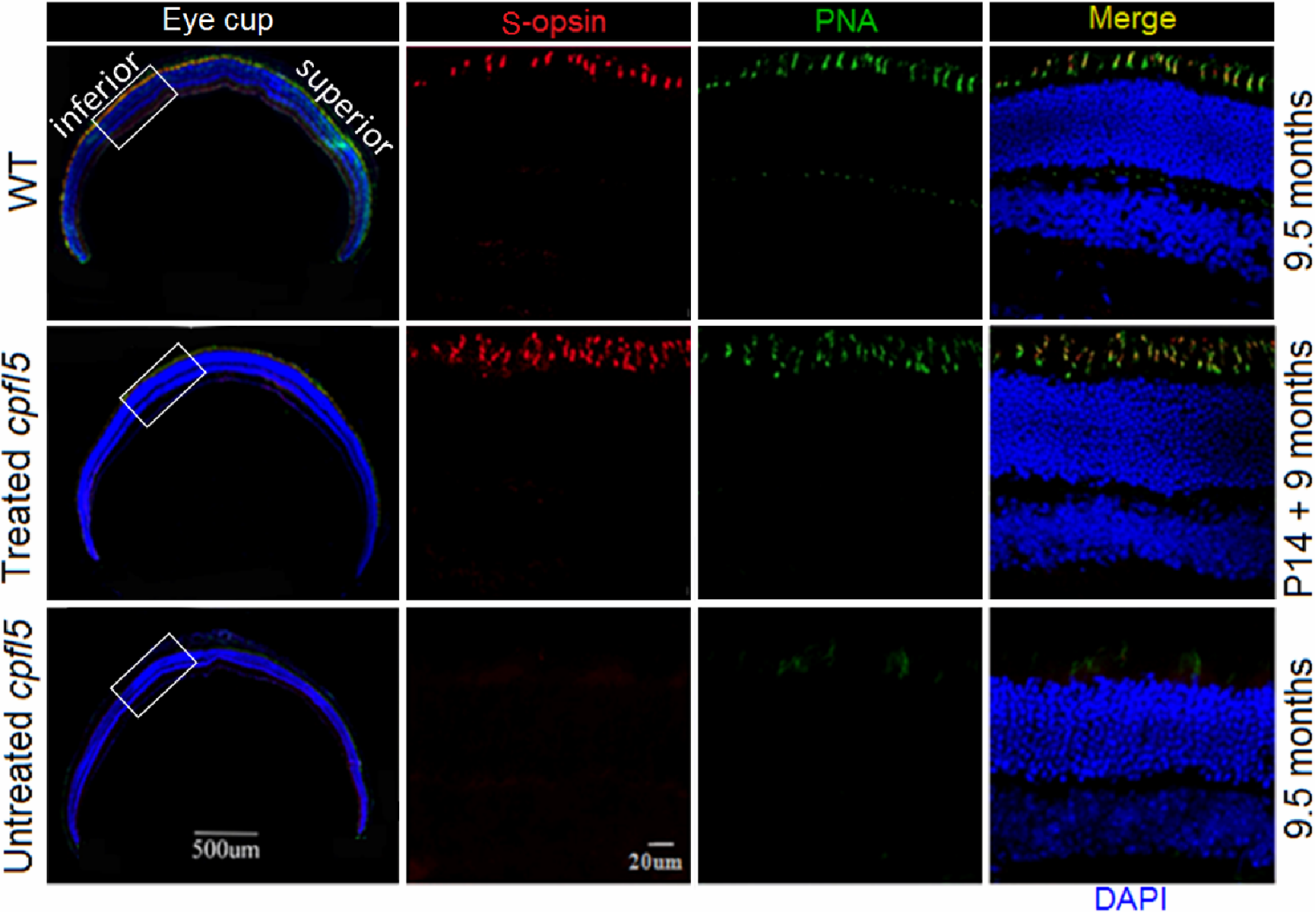
Long-term preservation of retinal S-opsin after treatment with AAV8 (Y447, 733F + T494V)-IRBP/GNAT2-*Cnga3*. At 9 months after treatment of P14 mice, *cpfl5* retinal immunostaining revealed normal expression (red) and the cone OS distribution (merge) of S-opsin compared with WT controls. S-opsin was not detected in untreated *cpfl5* retinas. In WT and treated *cpfl5* retinas, S-opsin was located in the cone OS. Red: S-cone opsin staining; Green: cone-specific PNA staining; Blue: nuclei staining with DAPI (4′,6-diamidino-2-phenylindole). P, postnatal day.

CNGA3 deficiency has been shown to impair expression and localization of cone opsins, ultimately leading to cone photoreceptor death in *Cnga3*^-/-^ and *cpfl5* mice [11,41]. Compared to age-matched WT retinas, untreated *cpfl5* retinas showed similar layers of photoreceptor nuclei and lengths of OS (Figs 5 and 6, right column). In untreated *cpfl5* cones, loss of S-opsin proceeded more rapidly than M-opsin [11]. By 9.5-months of age, no retinal S-opsin was detected (Fig 6, bottom row) and there were only a few residual cones in the superior retina with mislocalization of M-opsin (Fig 5, bottom row). We used PNA to detect the interphotoreceptor matrix sheath, which surrounds the cone OS. In treated *cpfl5* retinas, fluorescent microscopy at low magnification revealed preservation of M-opsin over most of the eyecup (Fig 5, second row). However, untreated retinas showed only some residual cones in which M-opsin had mislocalized to the inner segment, cone nuclei, and cone pedicles (Fig 5, bottom row). Double staining of M-opsin and cone-specific PNA (high magnification) suggests that the rescued M-opsin in treated retinas is located in the cone OS, consistent with that of WT controls (Fig 5, upper row). In addition, S-opsin was also in the cone OS of treated *cpfl5* retinas (Fig 6). In summary, AAV8 (Y447, 733F + T494V)-IRBP/GNAT2-*Cnga3* treatment at P14 maintained normal expression and distribution of cone opsins. Gene therapy using the AAV vector effectively prevented cone degeneration for at least 9 months.

**Fig 7:**
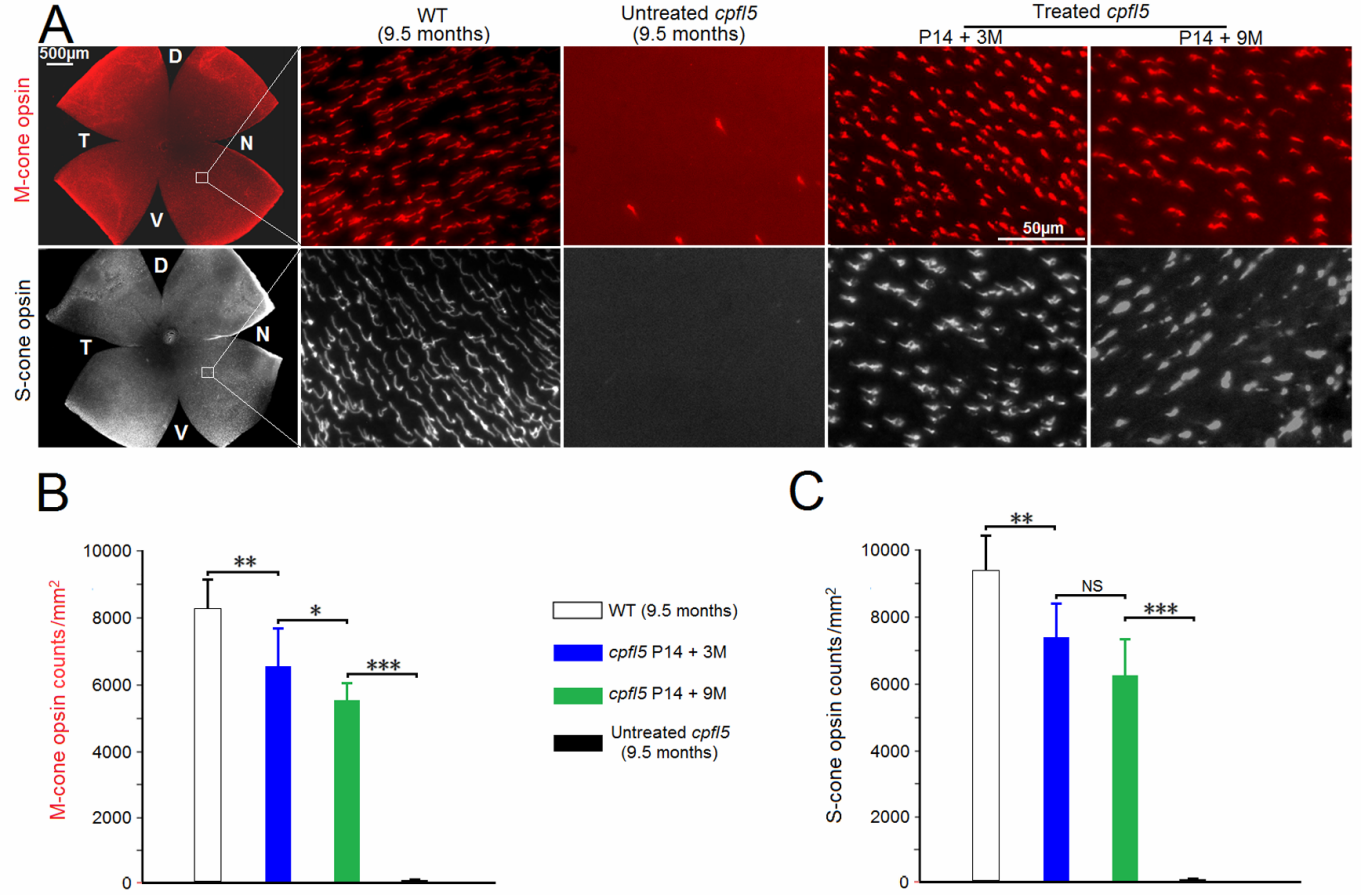
M-cone and S-cone opsins preservation in retinal whole mounts after treatment. At 3 and 9 months after P14 treatment with AAV8 (Y447, 733F + T494V)-IRBP/GNAT2-*Cnga3*, M-cone opsins (red) and S-cone opsins (white) were imaged (A) and counted (B, C) in the same field of ventral nasal retina (n = 6). WT and untreated *cpfl5* mice were used as controls. D, dorsal; V, ventral; T, temporal; N, nasal. P, postnatal day; M, months. *indicates *P* < 0.05, **indicates *P* < 0.01, ***indicates *P* < 0.001, NS = no statistical difference.

Retinal whole-mounts from the treated *cpfl5* eyes showed obviously preserved M-cone opsins and S-cone opsins compared with the contralateral untreated eyes (Fig 7A). At a high magnification (×40), the two cone opsins were counted in the same field of ventral nasal retina (Fig 7B and 7C). At 3 months after treatment, the average cone opsin counts were about 80% of WT levels (*P* < 0.01). However, M- and S-cone opsins decreased about 15% at 9 months following subretinal treatment. In the untreated *cpfl5* retinal whole-mounts, little M-cone or S-cone opsins expression was detected within the ventral nasal retinas.

### AAV8 (Y447, 733F + T494V) rescues cone-mediated water maze behavior in the *cpfl5* mouse

**Fig 8:**
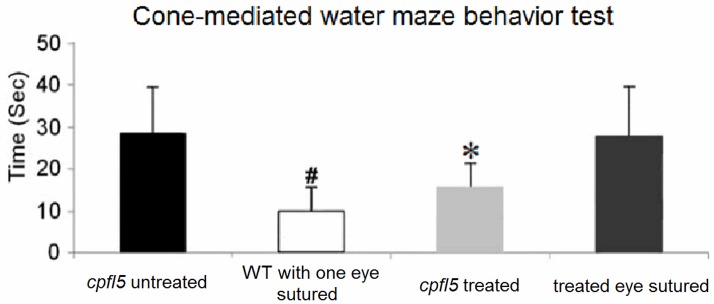
Cone-mediated visually guided behavioral test after treatment with AAV8 (Y447, 733F + T494V)-IRBP/GNAT2-*Cnga3*. ^#^Statistical analysis indicates a significant difference in performance (*P* < 0.01) in the WT mice with unilateral eyelid suture compared to untreated *cpfl5* mice and the treated *cpfl5* mice when the treated eye was sutured. *Statistical analysis indicates a significant difference in performance (*P* < 0.05) in the treated *cpfl5* mice compared to untreated *cpfl5* mice and treated *cpfl5* mice with the treated eye sutured. No statistical difference in performance was found between the WT mice with unilateral eyelid suture and treated *cpfl5* mice or between untreated *cpfl5* mice and treated *cpfl5* mice when the treated eye was sutured (n = 6).

To determine whether the observed electrophysiological, biochemical, and morphological preservation/restoration of the *cpfl5* retina following AAV8 (Y447, 733F + T494V) vector treatment led to improvement in behavioral performance, we tested mice in a visually guided behavior task (Fig 8), as described previously [40]. During formal tests, mice pupils were dilated and retinas were fully light-adapted to prevent rod intrusion. The time taken to escape to the platform under well-lit condition was compared among groups. The WT mice with unilateral eyelid suture took 10 ± 6 seconds, treated *cpfl5* mice took 16 ± 6 seconds, and untreated *cpfl5* mice took 29 ± 11 seconds to reach the platform. The data in each group were normally distributed (Shapiro-Wilk test, n = 6, *P* > 0.05). Statistical analysis showed significant improvement in the treated *cpfl5* mice compared to the untreated *cpfl5* group (n = 6, *P* < 0.05). No statistical difference in performance was found between treated *cpfl5* and WT mice (*P* > 0.05). When the treated eyes of *cpfl5* mice were closed by suturing their eyelids, time to reach the platform increased from 16 ± 6 to 28 ± 12 seconds. No statistical difference in performance was found between the untreated and treated *cpfl5* mice when the treated eyes were sutured (*P* > 0.05).

## Discussion

AAVs are non-pathogenic, single-stranded, DNA-packaging dependoparvoviruses within the Parvoviridae family. One of the major applications of AAVs is as a gene therapy vector to treat monogenic recessive blindness. For example, AAV serotype 2 has been adopted for ongoing RPE65-Leber congenital amaurosis (LCA2) gene therapy clinical trials [42]. Transgene expression efficiency of AAV vectors can be affected by phosphorylation of capsid surface-exposed residues, the phosphorylation ultimately leads to capsid ubiquitination and proteasomal degradation of AAV particles [31,32,43]. AAV2 variants containing capsid surface-exposed Y-F and/or T-V mutations protect vector particles from proteasomal degradation, thus significantly increasing the efficiency of retinal transduction [30,33,34,44]. Inhibiting proteasomal degradation may improve longevity of transgene expression. To test this, we investigated AAV variants from serotypes 2 and 8, the latter of which was recently reported to have higher photoreceptor transduction efficiency than wild-type AAV2 and 5 [45,46]. We compared rescue differences between three *cpfl5* groups, in which treatments were mediated by AAV8 (Y447, 733F + T494V), AAV8 (Y447, 733F), or AAV2 (Y272, 444, 500, 730F + T491V) vectors. At 1 month after treatment, photopic ERG responses were higher in AAV8 (Y447, 733F + T494V) and AAV2 (Y272, 444, 500, 730F + T491V) groups. Vectors with a combination of Y-F and T-V mutations seemed to lead to better ERG rescue than vectors with only a Y-F mutation. At 3 months after treatment, photopic responses of AAV2 (Y272, 444, 500, 730F + T491V)- and AAV8 (Y447, 733F)-treated retinas decreased, while the responses in those treated with AAV8 (Y447, 733F + T494V) were sustained or continued to rise slightly. Treated with the three AAV mutants, preservation of photopic-ERG responses was almost dose dependent across doses between 1E11 and 1E13 vg/ml. Among the original and diluted solutions, a dose at 1E13 produced the best improvement (Fig 1C and 1D). In summary, an AAV8 vector with both Y-F and T-V mutations gave the best preservation of cone ERG responses.

Besides design considerations to prevent proteasomal degradation, these results highlight the need for careful consideration of vector serotype in any therapeutic AAV vector platform. AAV8 vector can target a variety of retinal cells, including retinal pigment epithelial cells and photoreceptors, and its transfection is believed to be safe in mice, dogs and nonhuman primates [47]. Studies have showed that AAV8 has higher photoreceptor transduction efficiency than AAV2 and 5 [35]. Using WT or capsid mutant AAV8 vectors, gene therapy has successfully treated animal models of Leber’s congenital amaurosis, autosomal recessive retinitis pigmentosa and achromatopsia [35,45,46,48,49]. Compared to AAV2, AAV8 is able to achieve equivalent expression at lower dose [47,48]. In addition, transgene expression mediated by AAV8 is much sooner than AAV2 and 5 [45,47,48]. An AAV8 tyrosine-capsid mutant can confer more effective therapy than that of a standard AAV vector in an animal model with early-onset rapid retinal degeneration [45]. Besides AAV2, AAV8 can be another promising vector for human clinical gene therapy trials for choroideremia and even in the future for other retinal degenerative disorders [48]. Here, long-term Validation of capsid mutants AAV8 vector for CNGA3-achromatopsia has been demonstrated in a mouse model, but AAV vector performance in human patients could be different from mice. It is unlikely that concerns of long-term efficacy and safety can be sufficiently addressed in a small animal model. Thus, AAV8 or its mutants still need careful evaluation in large-animal models and/or non-human primates.

CNGA3-deficient retinas are characterized by early cone photoreceptor dysfunction, followed by rapid S-opsin loss, abnormal M-opsin distribution, and ultimately, cone cells death [11,41]. Three-week-old *cpfl5* retinas exhibit normal M-opsin staining in the cone OS, but already show a decrease in S-opsin [11]. By 10 weeks, little S-opsin staining is detected and all M-opsin has mislocalized to the cone inner segment, nuclei, and pedicles [11]. As to the 9.5-month-old *cpfl5* mice, we found a loss of the mislocalized M-opsin in the inferior retinas (Fig 5).

Here, gene therapy through subretinal injection was performed at P14 to achieve maximum rescue of cone photoreceptors. In mice younger than P14, especially within one week after birth, trans-corneal subretinal injection could cause severe damage to cornea, iris, lens, and retina because of the smaller eyeballs, underdeveloped cornea and lens, and difficulty in achieving excessive dilatation of the pupil, as described previously [36]. Additionally, it is difficult to detach a significant fraction of the mouse retina via subretinal injection prior to P14 [38]. We have previously found that subretinally injected vectors localized around the injection area in P10 mice and ultimate coverage of the vector solution was no more than one-third of the whole retina, an area much smaller than P14 injections [38].

AAV vectors containing the IRBP/GNAT2 promoter exhibit cone photoreceptor-specific transgene expression [35]. Using AAV8 (Y447, 733F + T494V)-IRBP/GNAT2-*Cnga3*, we obtained a more robust rescue than using AAV2 (Y272, 444, 500, 730F + T491V) or AAV8 (Y447, 733F) vectors in *cpfl5* mouse, a spontaneous animal model of achromatopsia with a *Cnga3* mutation. Photopic ERG responses in the mouse retina were rescued and maintained to an average of 2/3 of the WT mouse responses at 9 months after injection of AAV8 (Y447, 733F + T494V). Moreover, the rescued cone-ERG responses showed no implicit time delay. These data are from the longest follow-up period reported thus far. In our previous work, the long-term cone-ERG responses of a WT AAV5 vector treatment were significantly lower than those of the AAV8 triple-mutant vector treatment. The water maze visually guided behavioral test has been used in dark conditions to examine AAV-mediated rod function recovery in *rd12* mice [40]. Here, we extended this application to test cone function in pupil-dilated and fully light-adapted *cpfl5* mice. In addition to functional outcomes, our data show biochemical and structural preservation/restoration following AAV8 (Y447, 733F + T494V) vector treatment.

Cone-mediated ERG responses decreased about 20% from 3 to 9 months after treatment with the AAV8 (Y447, 733F + T494V) vector. In accordance with the deterioration of cone-ERG responses, the two cone opsins decreased about 15% at 9 months. This is likely due to inadequate expression of CNGA3 in some cone photoreceptors. Relative to 3 months following AAV8 (Y447, 733F + T494V) treatment, the immunostaining of rescued CNGA3 seemed to be lesser and weaker at 9 months (Figs 3 and 4). Additionally, the decrease upon treatment may be partly caused by a damaging effect of subretinal injection. In human retinas, cone density is very high in the central macula. Retinal detachment of the fovea, caused by subretinal injection, may cause more damage than benefit [30]. Rod-mediated ERG responses showed a reduction of b-wave amplitudes in treated compared with untreated eyes, similar as described previously [11]. The decrease upon treatment may be a consequence of injection-related damage.

Recently, successful gene therapies have been reported in CNGA3-deficient sheep, a large-animal model of achromatopsia [50–52]. In these studies, AAV serotype 2 and 5 vectors carry *Cnga3* gene under control of a human red/green cone opsin promoter. The results of a large-animal model provide an important data base for gene therapy in CNGA3-achromatopsia patients.

Here, we show that gene therapy mediated by AAV8 (Y447, 733F + T494V)-IRBP/GNAT2-*Cnga3* can effectively rescue cone degeneration in the retinas of *cpfl5* mice for at least 9 months. To our knowledge, this is the longest and most significant rescue reported so far in the mouse model of *Cnga3*-associated achromatopsia.
